# Hydrogel Adsorbents for the Removal of Hazardous Pollutants—Requirements and Available Functions as Adsorbent

**DOI:** 10.3390/gels8040220

**Published:** 2022-04-03

**Authors:** Yoshimi Seida, Hideaki Tokuyama

**Affiliations:** 1 Natural Science Laboratory, Toyo University, 5-28-20 Hakusan, Bunkyo-ku, Tokyo 112-8606, Japan; 2 Department of Chemical Engineering, Tokyo University of Agriculture and Technology, 2-24-16, Naka-cho, Koganei, Tokyo 184-8588, Japan; htoku@cc.tuat.ac.jp

**Keywords:** polymer hydrogel, adsorbent, adsorption, hazardous pollutant, network function

## Abstract

Over the last few decades, various adsorption functions of polymer hydrogels for the removal of hazardous pollutants have been developed. The performance of hydrogel adsorbents depends on the constituents of the gels and the functions produced by the polymer networks of the gels. Research on hydrogels utilizing the characteristic functions of polymer networks has increased over the last decade. The functions of polymer networks are key to the development of advanced adsorbents for the removal of various pollutants. No review has discussed hydrogel adsorbents from the perspective of the roles and functions of polymer networks in hydrogels. This paper briefly reviews the basic requirements of adsorbents and the general characteristics of hydrogels as adsorbents. Thereafter, hydrogels are reviewed on the basis of the roles and functions of the polymer networks in them for the removal of hazardous pollutants by introducing studies published over the last decade. The application of hydrogels as adsorbents for the removal of hazardous pollutants is discussed as well.

## 1. Introduction

Conservation of water environments and water resources is a critical issue of this century. Efficient purification technology to reduce water pollution is essential, along with the promotion of reduced emission of pollutants. In the treatment and remediation of large water bodies, adsorption is an effective method for the separation of low-concentration pollutants. In the treatment of wastewater containing hazardous substances, the adsorption method is also a candidate process owing to its simplicity and cost-effectiveness. Various cost-effective solid adsorbents such as zeolites, activated carbon, modified clays, and ion-exchange resins have been developed for water treatment. Inevitably, the efficiency of the adsorption process relies on the performance of the adsorbent. Organic polymer hydrogels comprising a three-dimensional polymer network and a solvent have been shown to act as adsorbents for heavy-metal ions, dyes, and other organic hazardous pollutants because of their high performance, simplicity of use, and diversity in adsorbent design. Over the last few decades, the development of hydrogel adsorbents that effectively utilize the functions of polymer networks has increased [1,2,3,4,5,6,7]. When utilizing hydrogels capable of comprehending various functions as advanced adsorbents, it is essential to design their functions to meet the required objective. The characteristic functions of the gel form are due to the cooperative effect of the physicochemical properties of the gel constituents and the functions provided by the polymer network of gels. Many reviews of gel adsorbents are extensively available in the literature, which focus on their adsorption capacity as a function of the chemical structure, morphology, and adsorption characteristics of the gels. However, no review has discussed the performance of gels based on the roles and functions of the polymer network of gels, although this viewpoint will be effective in the development of advanced hydrogel adsorbents. Classification of hydrogels, focusing on the roles and functions of the polymer network, may provide inspiration for the adsorbent design of advanced hydrogels. It is also essential to understand the basic requirements of adsorbents in advance to examine hydrogels as adsorbent candidates.

In this short review, the basic requirements for adsorbents and the general advantages and challenges in the application of hydrogels as adsorbents are first reviewed. Then, the characteristics, advantages, and subjects of hydrogels as adsorbents are reviewed based on the roles and functions of the polymer network of gels, with an overview of studies published in the last decade. This review offers engineers and researchers an opportunity to gain more knowledge on hydrogels as adsorbent candidates.

## 2. Basic Requirements for Adsorbents

The design and use of adsorbents in the adsorption separation process are essential for adsorption separation technologies. Adsorbents that meet requirements like material cost, minimal environmental impact, stability, uptake rate, reusability, and adsorption capacity for target adsorbates will be selected in practice. In the modern design of adsorbents, the physicochemical structure of the adsorbent can be tailored to target pollutants. In the design of the adsorption separation process, the parameters of both the adsorption equilibrium and the mass transfer in adsorbents are crucial [8,9]. The adsorption isotherms, the curves of semi-empirical adsorption equilibrium showing the adsorption capacity as a function of adsorbate concentration, represent the performance of the adsorbent. Adsorbent users can estimate the performance of adsorbents under the condition of use based on the adsorption isotherms and relevant conditions. Therefore, adsorption isotherms are indispensable for the practical use of adsorbents, although data acquisition of adsorption isotherms is time-consuming. The adsorption isotherm or adsorption capacity of adsorbents depends on a range of factors such as temperature, ionic strength, solvent composition, particle size, and adsorber operation [8,9]. In the design of adsorption processes and evaluation of adsorbents using literature data of adsorption isotherms or capacities, it is necessary to confirm the conditions under which the adsorption data in the literature were collected, to evaluate the adsorption process properly. It is also essential to consider desorption and regeneration methods involving simple operations and minimal waste. Because extra solvent wastes are produced in the recovery and regeneration processes, the desorption process should also be optimized.

The typical approach for improving the adsorption capacity is micronization and increasing the specific surface area of the adsorbents. Fibrization of adsorbents, as observed in non-woven fabrics, has also been used in recent years [10,11]. A considerable increase in accessible meso- and micro-sized pores in the adsorbents is found to increase the effective surface area. The percolated porous structure of the adsorbents reduces the mass transfer resistance inside the adsorbents, leading to the effective utilization of the adsorption sites. Simple methods for the determination of the mass transfer parameters of adsorbents have also been reported for a series of adsorber reactor types [12,13,14,15]. 

From an adsorption engineering point of view, the adsorption performance depends on operational conditions such as the adsorber reactor type, solid–liquid ratio, and solution–adsorbent contact time [8,9]. When the adsorbents are used in a packed column, the smaller the particle size the larger the flow resistance. At the industrial level, with high throughput, considerable flow resistance imposes economic and operational burdens. Thus, porous adsorbents are effective from this perspective. When adsorbents are used in batch systems, the boundary layer resistance of the adsorbents and solid–liquid separation for the recovery of adsorbents are issues to be considered for simple and rapid adsorption separation [15], especially in the case of fine adsorbent particles. The general idea is that an excellent combination of the adsorbent and optimum process operation results in the best adsorptive separation performance. If either is inferior, adequate adsorptive separation performance cannot be obtained.

## 3. Gel Adsorbents

Some adsorbents include a local gel phase. In most cases, only an adsorbent in which the entire adsorbent consists of a gel phase is called a gel adsorbent. Polymer hydrogels have several advantages in their application as adsorbents. The polymer networks create percolation pores with the desired solvent content and pore size. The specific surface area of polymer hydrogels is not typically discussed because of their network structure with a large amount of solvent. Open pores created by the polymer networks in the gels enable adsorbates to percolate deep inside the gels quickly, thus facilitating the adsorption capacity of the gels. Gels with high solvent content exhibit excellent diffusional permeability toward a range of substances. The diffusivity of solutes in gels has been extensively studied, both experimentally and theoretically. Various mathematical models used to determine the diffusion coefficient in gels have been developed and summarized in studies published in the 1990s [16,17]. Nevertheless, the study of diffusivity remains an interesting scientific topic of discussion [18,19,20,21,22,23,24]. The development of porous structures in polymer gels can potentially increase the number of effective adsorption sites and reduce mass transfer resistance. Many methods for synthesizing porous hydrogels, such as phase separation [25,26,27], emulsion [28,29,30,31], ice crystal [32], and foam [33,34], have been reported. A solution environment that is different from the outer solution can be created inside the gels depending on the nature of the polymers, such as the hydrophilic–hydrophobic balance of polymers and the existence of fixed charges in the polymers. A gel liquid-phase system with an arbitrary size, interface, and less solvent disturbance is unique and valuable. One of the significant advantages of particulate gels is their ease of liquid separation for solvent extraction. The solvent extraction phase can be immobilized on the gels; rendering the handling of solvent easy. When stimuli-responsive polymers are used, the structure and physical properties of the gel can be reversibly controlled by the stimuli to which the polymers respond. The stimuli-responsive property of the gel produces a dynamic shift in the adsorption isotherm [35], which is effective in adsorptive separation systems. The network structure of gels can create integrated reaction fields, characteristic of polymer hydrogels formed by a three-dimensional polymer network and immobilized solvent, as mentioned above. The characteristics and functions of the polymer network include open pores with a large pore volume, a scaffold for functional group immobilization, compatibility with solvents, stimuli-responsiveness, strength imparting, and a specific environment different from the outer solution in terms of pH, ionic strength, and redox potential.

However, hydrogels have some drawbacks. Mechanical strength is a significant issue in many applications of the gels. The mechanical strength is an essential factor for the practical use of hydrogels. Thus, the strength of hydrogels has been improved by polymer selection and the structural design of polymer networks, such as the use of stiff polymers, an increase in the degree of polymerization, and the crosslinking method. Composites have successfully resolved the subject of strength through immobilization in a stiff porous matrix, the introduction of fiber materials such as nano-cellulose [36,37,38], porous networks [28,34], and double networks [39,40,41]. Cost reduction is also required in many adsorption separation applications, especially in applications with large treatment volumes, as seen in sewage treatments. Because synthetic gel adsorbents are economically less competitive than conventional adsorbents such as zeolites and activated carbons, gel adsorbents are expected to be competitive with processing simplicity, superiority over conventional adsorbents, and some other advantages. Conventional adsorbents can be substituted for gel adsorbents when the candidate adsorbents are economical and readily available for practical use, even if the adsorption performance is not optimal and the adsorbents are not highly sophisticated. Therefore, apart from academic interest, gel adsorbents are expected to be easy to use, high performance, cost applicable, and will need a reason that the adsorbent should be the gels. Economic benefits can be gained using natural products and unused biomass. There are few reports of these studies at a practical level. Reproducibility, accuracy, and longevity are also essential factors in hydrogel adsorbents with stimuli-responsive functions. From this perspective, the advantage of gel adsorbents is the characteristic function created by the polymer network with the solvent.

Applications in which these functions are suited have been explored in many fields. Studies reviewing gel adsorbents in the last decade are introduced as follows.

Naseem et al. (2018) reviewed microgels as adsorbents for the removal of aqueous pollutants such as heavy metals, nitroarenes, organic matter, and toxic dyes [6]. The microgels were classified based on their synthesis methods, response behaviors, and compositions. The development of microgel usage and the operational factors that influence the adsorption and recovery performances are discussed, along with the cost and environmental impact of the microgels. Tran et al. (2018) reviewed hydrogels with entrapped particles, focusing on the shape of the gels, including hydrogel beads, films, and nanocomposites [3]. Their application in the adsorption of contaminants in water and wastewater treatment has been introduced. Samaddar et al. (2019) reviewed hydrogels based on their composition and functional groups [5]. The hydrogels were classified into six classes: polymeric hydrogels synthesized from vinyl monomers, polymeric hydrogels with amine and amide groups, polysaccharide gels, graphene-embedded hydrogels, nanomaterial-embedded hydrogels, and biomolecule-embedded hydrogels. The characteristics, applications, and adsorption properties of pollutants were investigated for each gel class. The adsorption mechanism, adsorption efficiency, and factors affecting adsorption efficiency are discussed. Akhmetzhan et al. (2021) reviewed recent studies on N, N-dimethylacrylamide (DMAA)-based hydrogels for the removal of cationic dyes and heavy-metal ions from water [7]. Methods for increasing the adsorption capacity for heavy-metal ions are discussed. Copolymerization of hydroxyethyl methacrylate and inclusion of graphene oxide with tragacanth gum, by which the sites interacting with adsorbates were increased in the gels, resulted in a substantial increase in the adsorption capacity.

Many studies on natural polymer gels as adsorbents have been reported. Various adsorbents using natural polymers and biomass, such as alginate [42,43], chitosan and its derivatives [44,45,46], silk [47], cellulose and its derivatives [2,48,49,50], tannin [51,52,53], pectin [54,55], cotton [39], rice husk [40], algae [56], aloe vera [57], cyclodextrin [58], and their conjugates [41,43,59,60,61,62,63], have been synthesized, and their pollutant adsorption characteristics have been reported. Inoue et al. (2017) reviewed biosorbents (lignophenol gel, seaweed waste, fruit juice residues) for remediating aquatic environmental media polluted with hazardous heavy metals and metalloids [54]. Francis et al. (2021) reviewed chitosan-based gel adsorbents and discussed the adsorption mechanism of gels for Cr(IV), Cd(II), phenol, and methylene blue [46]. Qi et al. (2021) reviewed recent advances in polysaccharide-based adsorbents, providing general design principles for polysaccharide-based adsorbents, critical factors that may affect their adsorption, and future directions for the development of polysaccharide-based wastewater adsorbents [64]. 

Lim et al. (2019) reviewed molecular gels as adsorbents for environmental remediation and water treatment and indicated the advantages of molecular gels with large selective capacities for pollutant sorption and recovery [65]. Molecular gels consist of self-assembled fibrillar networks and are distinguished from gels made of polymers (polymeric gels). They introduced recent developments in molecular gels for oil-spill remediation, removal of heavy metals from wastewater and contaminated water, anion remediation, and removal of dyes by the molecular gels. Several subjects, such as their mechanical properties and recyclability in applications, were also discussed. 

Hande et al. (2015) reviewed ion-imprinted polymer gels (IIPs) [66]. Methodologies for the synthesis of IIPs and heavy-metal removal and monitoring applications are also discussed. They evaluated the methodologies and monitoring performance of IIPs based on metal ions, ligand/complexing monomers, adsorption capacities, and relative selectivity coefficients. Imprint gels are superior to ion-exchange resins in terms of their selectivity and economy. However, there is still a need for the design and development of gels with improved adsorption rates and surface areas. 

Samiey et al. (2014) reviewed organic–inorganic composite adsorbents for heavy-metal ions based on the composite structure of the adsorbents [67]. The merits of the composites are discussed, and the advantages of natural polymers (humic acid, chitosan, cellulose) as combined organics are indicated. Claverie et al. (2019) reviewed organic-inorganic hybrid polymers for the removal of heavy metals [68]. Methodologies for the synthesis of porous, layered, hierarchical, and interpenetrating–structured hybrid polymers are introduced. The adsorption performance was summarized as a function of the interacting chemical groups, type of adsorption isotherm, and adsorption capacity of each hybrid adsorbent. Maleki et al. (2016) reviewed aerogels as adsorbents for heavy metals and organic pollutants such as dyes, oils, VOCs, and heavy-metal ions [69]. Many unclear points about the desorption mechanism of heavy metals in the aerogels have been indicated, and the need for further research on regeneration and desorption methods is proposed. Wang et al. (2019) reviewed recent advances in the electrochemical sensing of heavy-metal ions based on electrodes modified with polymers such as conducting polymers, polymer composites, and ion-imprinted polymers [70]. The merits of polymer-based electrochemical sensors, in terms of cost, sensitivity, selectivity, and stability are discussed. As described above, many reviews have classified gel adsorbents based on their morphology and chemical composition (materials) and discussed the adsorption characteristics of heavy metals and dyes.

## 4. Classification of Hydrogels Based on the Roles or Functions of the Polymer Networks

Polymer gels as adsorbents were classified based on the roles and functions of the polymer networks in their applications as follows. 

### 4.1. Matrix for Immobilization

The simple use of hydrogels is for the immobilization of supports and carriers. Even in this simple application of hydrogels, they have various advantages. The incorporation of diverse materials, simple solid–liquid separation, stabilization of the immobilized substances maintaining high dispersion, accumulation and enrichment of target substances inside the gels, and the size control of stable solvent droplets with the size of gels are distinct advantages of the gels. The difference between hydrogels and emulsions or liquid immobilizing capsules is that water is immobilized in the polymer network with percolating open pores, and mass transfer across the surface of the gels is not restricted. The size of gels and pores are controllable, and mass transfer in the gels is not reduced, owing to the large amount of water and the percolating open pores in the hydrogels, which are also effective for the immobilized microorganisms and macromolecules, such as antibodies and enzymes, to maintain their activity over a long time. Furthermore, gels can produce a hierarchical reaction field because zoning of embedded substances can be achieved owing to the immobilization property of the polymer network.

**Figure 1:**
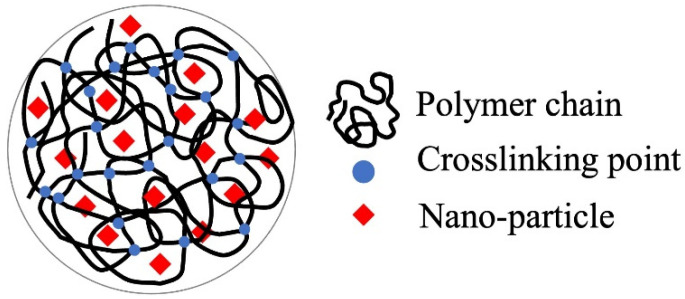
Hydrogel for the immobilization support and carrier.

**Figure 2:**
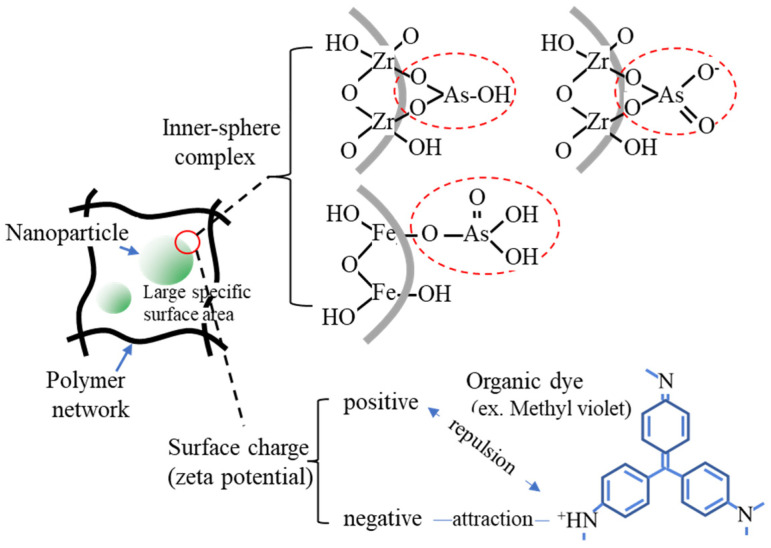
Examples of the adsorption mechanism on the nanoparticles.

The immobilization of fine particles in gels enables easy handling of fine particles in the liquid-phase adsorption process. This advantage of gels is significant in adsorption separation operations in batch processes, especially in the use of nano- and microparticles as adsorbents. The polymer network of the gel enabled the highly dispersed immobilization of nanoparticles (Figure 1), which contributed to the increase in the effective surface area of the nanoparticles. Nanoparticles can be embedded by the polymerization of gel monomers in the suspension of nanoparticles or by their synthesis inside the gel matrix. Various composite gels immobilizing nanoparticles have been reported in the last decade, including amidoximated poly(methacrylic-*co*-acrylonitrile) composite gel immobilizing magnetic CoFe nanoparticles for the removal of Cr^3+^, Cd^2+^, and dyes [71], Fe_2_O_3_ nanoparticles immobilizing polyacrylamide/chitosan composite hydrogel for the removal of methylene blue [72], hydrous ferric oxide immobilizing poly(trans-Aconitic acid/2-hydroxyethyl acrylate) hydrogel for the removal of Pb^2+^, Cu^2+^, Cd^2+^, and Ni^2+^ [73], zirconia nanoparticles immobilizing poly(N, N-dimethyl acrylamide) hydrogel for the removal of As_2_O_3_ and AsO_4_^3−^ [74], and iron hydroxide (lepidocrocite) immobilizing poly(N, N′-dimethylaminopropyl acrylamide, methyl chloride quaternary) cationic hydrogel for the removal of AsO_4_^3−^ from groundwater [75]. These gels increased the adsorption capacity by combining them with nanoparticles. Some examples of the adsorption mechanisms of nanoparticles are illustrated in Figure 2 [75,76,77]. The adsorption mechanism is classified into inner-sphere surface coordination complexes (strong bonding), outer-sphere ion-pair complexes, and electrostatic interactions. As_2_O_3_ and AsO_4_^3−^ were adsorbed on the zirconia and iron hydroxide nanoparticles via the inner-sphere complex. The large surface area and surface charge of the nanoparticles would also contribute to the adsorption capacity. From this perspective, composites with metal nanoparticles produced positive adsorption results. Bimetallic nanoparticles, such as CoFe, NiFe, and CuNi, have been studied in water treatment for the removal of heavy metals and decomposition of dyes from aqueous solutions owing to their specific adsorption behaviors for pollutants, although the mechanism of adsorption on the nanoparticles has not been extensively studied [78,79].

Conventional solvent extraction methods involve the extraction of specific metal ions from an aqueous solution into an organic solvent phase containing an organic extractant (an oil-soluble complexing agent). Hydrogels can retain oil microdroplets. Hydrogels that immobilize oil microdroplets can be prepared using the emulsion–gelation method [28]. The gels were obtained by the free-radical polymerization of gel monomers in the aqueous phase of an oil-in-water (O/W) emulsion. The emulsion gels with the oil phase enabled the extraction of targets from the outer solution into the gels via a solvent extraction mechanism. The extraction of In(III) and Zn(II) using poly[poly(ethylene glycol) methyl ether acrylate] emulsion gels and the extraction of Pd(II) have been reported [80,81].

In petroleum resource recovery, hydrophilic gel particles are used for enhanced oil recovery. The gel particles absorb water to increase the viscosity of the aqueous phase in the oil reservoir, thereby reducing the liquidity of the water phase and facilitating oil recovery. Hydrogels are also used for oil/water separation from oil-containing wastewater exhausted from the oil industry by selective water absorption and permeation function of the hydrogel. Studies on poly(acrylamide-*co*-acrylic acid), poly(lauryl methacrylate), cellulose, alginate, and guar gum have been reported [82]. Hydrogels containing amine-based deodorants are used to absorb odorous components. The water in the gels enhances the adsorption of odorous components from the air, and the absorbed odorous components are chemically immobilized in the amine groups [83].

### 4.2. Scaffold Fixing Functional Groups

A polymer network is the backbone of hydrogel adsorbents. Functional groups that function as active sites for the adsorption of pollutants are immobilized on the polymer network. The functional groups can be freely introduced into the polymer network, and the solvent content can be adjusted intentionally in hydrogel adsorbents. Thus, the basis of adsorbent design is to use the functional groups that interact with the target adsorbates in preference. Polymeric gel adsorbents can be prepared by polymerizing monomers with desired functional groups or by post-chemical modification of the polymer network to obtain the desired functional groups. Increasing the adsorbent’s surface area and grafting the branched polymer onto the backbone polymer chains are generally used to increase the number of effective adsorption sites in the adsorbents, as mentioned above. Most studies focus on the adsorption performance, but not the adsorption mechanism, despite the importance of the adsorption mechanism in practical process control. The interactions facilitating adsorption are determined by the type of adsorbate, functional groups involved in the adsorption with their spatial layout, and solution conditions such as pH and salinity. In the case of rigid polymers such as chitosan, for instance, the conformation of the chitosan polymer and pH-dependent charge of the amine groups in chitosan affect the coordination bonding between heavy-metal ions and the functional groups of the chitosan (amine groups and hydroxyl groups) [44]. Representative synthetic monomers and natural polymers are shown in Table 1 along with the functional groups attached to them [48,53,54,64,84,85]. The use of functional groups that interact with potential adsorbates via ion exchange, electrostatic interactions, van der Waals forces, chelation, or hydrophobic interactions is essential. Gels with anionic groups are used to remove heavy-metal cations and cationic dyes, and gels with cationic groups are used for heavy-metal anions and anionic dyes. The typical weak acid groups are carboxyl groups, the strong acid groups are sulfonic groups, and the typical base functional groups are amine groups. For example, acrylic acid, acrylamide 2-methylpropane sulfonic acid (AMPS), and vinyl amine are used to impart weak acids, strong acids, and weak base groups, respectively. For metal ions, hydrogels with chelate-forming functional groups have also been used. Substances with electron-donor groups form specific coordination bonds with target metal ions such as vinylpyridine, N-vinyl imidazole, polyethyleneimine, dithiocarbamate, and methacryloylhistidine, depending on the target metal ions. Thus, selective adsorption can be achieved using functional groups that specifically adsorb metal ions in chelate-forming hydrogels. It should be noted that the solution pH, salinity, and other contaminated ions are disturbing factors that influence the adsorption capacity of ionic gels. Polymers with hydrophobic moieties, such as phenyl groups and long alkyl chains, are commonly used in the adsorption of organic pollutants through hydrophobic interactions. Amphiphilic groups that interact with the hydrocarbon groups of oil are candidate functional groups for oil adsorption. Sada et al. (2017) developed lipophilic polyelectrolyte gels consisting of polystyrene with a small number of lipophilic electrolytes derived from triphenylphosphonium tetraaryl borates. The triphenylphosphonium group is superior to the alkyl ammonium group and is responsible for the expansion of the range of applicable organic solvents [86]. A design that makes the gels porous and quickly absorbs adsorbents is essential for oil adsorbent gels [87]. 

The use of abundant natural polymers and unused biomass has increased owing to their economic advantages and the need for sustainable green processes in adsorption technology. Water-soluble natural polymers are crosslinked to obtain insoluble gels for easy handling. In the gelation of water-soluble natural polymers, adsorption sites, such as amino groups, are used for crosslinking, reducing the adsorption capacity of the adsorbents [1]. Thus, the gelation method is an important choice for achieving the best performance of the adsorbent in the case of natural polymers. 

Many hydrogels, including poly(acrylic acid) [88], poly(*N*-isopropyl acrylamide) [89,90,91,92,93,94], poly(*N*,*N*-dimethylacetamide-*co*-2-hydroxyethyl methacrylate) [95], amidoximated poly(methacrylic-*co*-acrylonitrile) [71], poly(2-hydroxyethyl methacrylate)-grafted copolymer [96], poly(glycidyl methacrylate-glycine) [97], poly(1-vinyl-2-pyrrolidone-*co*-sodium acrylate) [98], and poly(maleic acid) with cyclic groups of poly (1, 4-dioxa-7, 12-diazacyclotetradecane-8, 11-dione) and poly (1,4-diazocane-5,8-dione) [99,100] have been studied in the last decade to investigate their adsorption properties over various targets. As typical adsorption characteristics of hydrogels, Sammaddar et al. (2019) [5] and Qi et al. (2021) [64] reviewed the adsorption capacity of the hydrogel adsorbents such as acrylic acid, amine, alginic acid, cellulose and its derivatives, pectin, and chitosan gels for the removal of pollutants from the aqueous system. In addition to these hydrogel adsorbents, hybrid and composite hydrogels involving natural polymer blend composites and clay/natural polymer composites have been synthesized, and their adsorption properties for hazardous pollutants have been reviewed [63,101,102]. Shalla et al. (2018) reviewed hydrogel composites for the removal of recalcitrant organic dyes, including synthetic polymer-natural polymer, clay polymer, graphene polymer, and metal oxide–polymer composites [4]. As ionic dyes adsorb via electrostatic interactions, it is essential to increase both the number of adsorption sites and their dispersibility using materials with a high affinity for dyes in the composites.

### 4.3. Stimuli-Responsive Network

Thermoresponsive hydrogels are representative adsorbents of this type. These gels reveal a change in the hydrophilic–hydrophobic balance of the polymer network depending on the temperature. This property is often observed in amphiphilic polymers. Representative amphiphilic polymers include poly(*N*-isopropyl acrylamide), poly(*N*-substituted acrylamide), and their copolymers. Polymers with a lower critical solution temperature (LCST) in an aqueous system exhibit hydrophilicity with hydration below the LCST. At temperatures above the LCST, the polymers exhibit hydrophobicity with dehydration. This property enables gels to adsorb and desorb organic molecules with hydrophobic moieties through temperature control across the LCST [35].

**Figure 3:**
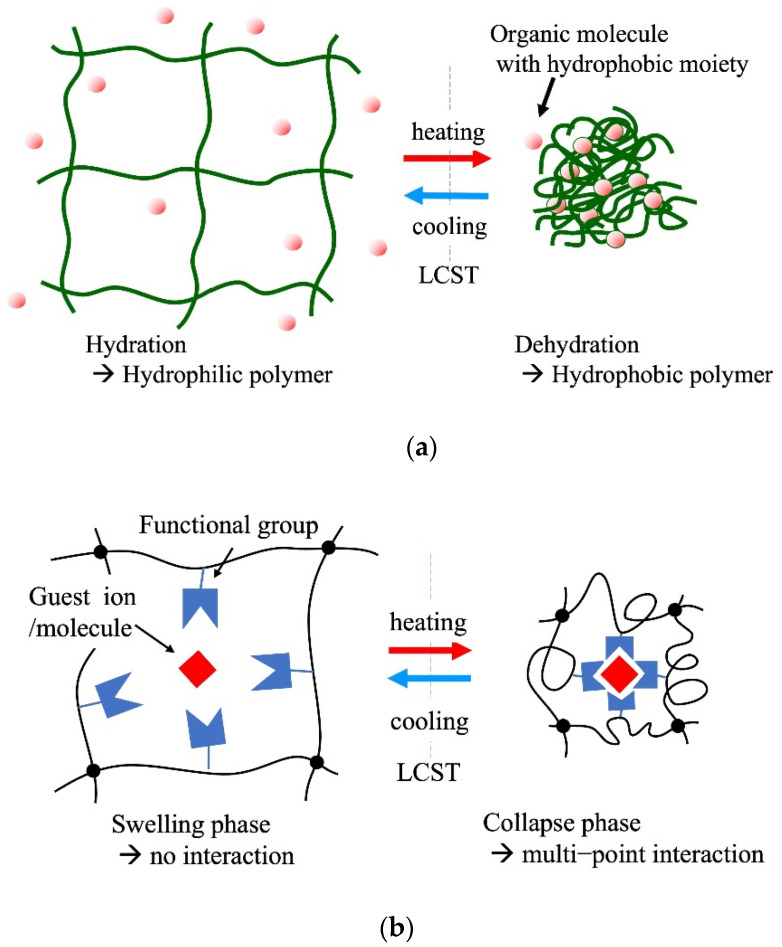

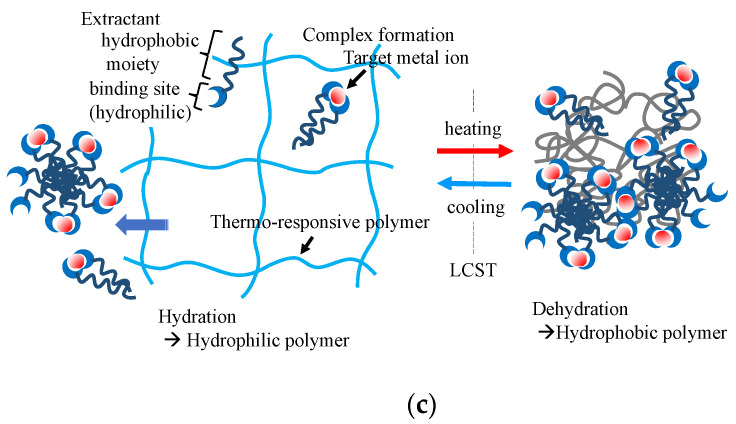
Thermoresponsive adsorption control using amphiphilic polymer hydrogel in aqueous systems, (**a**) adsorption through hydrophobic interaction between the thermoresponsive hydrogel and the molecules with hydrophobic moieties, (**b**) selective multipoint interaction, (**c**) solid-phase extraction of metal ions.

Figure 3a shows the basic concept of temperature-controlled adsorption and the separation of organic contaminants in water using thermoresponsive hydrogels. The critical temperature of the adsorption–desorption control corresponded to the LCST of the polymers used in the gels. LCST can be controlled by the copolymerization of a small number of co-monomers that differ from PNIPA in terms of hydrophobicity. Various thermoresponsive copolymer hydrogels, such as *N*-isopropyl acrylamide (NIPA)-*co*-acrylamide, NIPA-*co*-polyacrylic acid (PAA), NIPA-*co*-dimethyl acrylamide (DMA), and NIPA-*co*-acryloyl piperidine, have been synthesized, and their thermoresponsive adsorption properties for organic molecules have been reported in the 1990s. The adsorption amount of adsorbates in this system depends on the difference in the hydrophobicity of the adsorbates. This principle of adsorption control was applied to the chromatographic separation of steroids in 1996 by Kanazawa et al. (1996) [103]. This chromatographic separation process in the aqueous phase demonstrated organic-eluent-free separation. Changes in the volume of the gel occur along with the control of the hydrophilic–hydrophobic balance in this system. Therefore, when applying the gel to column separation, it is necessary to devise ways to use it, such as immobilizing the gel in a porous support. The stimuli to which the temperature-responsive gels respond can be diversified by copolymerizing a small amount of a second stimulus-responsive monomer into the gel [104]. 

Figure 3b shows the self-regulated multipoint adsorption. This system adsorbs target substances through multipoint interactions with functional groups on the polymer network. In the swelling phase of the gel, the functional groups hardly interact with the target at a stable interaction distance and the number of functional groups controls the swelling of the polymer network. In the collapsed phase of the gel, the functional groups could access the target and adsorb multiple interaction points at each stable distance. Thermoresponsive adsorption of this type was demonstrated using an *N*-isopropyl acrylamide (NIPA)-*co*-4-(vinylbenzyl) ethylenediamine (VBEDA) gel for Cu(II) removal [105]. In the chelating function, soft ligands that interact weakly with metal ions are useful for adsorption control based on the swelling volume and ligand spatial density in stimuli-responsive gels.

Tokuyama et al. (2007) reported solid-phase extraction of Cu(II) using a poly(*N*-isopropyl acrylamide) hydrogel [90]. Cu(II) complexes with their extractants or micelles, which are formed using an anionic surfactant such as sodium n-dodecylbenzene sulfonate and n-dodecyl benzene sulfonic acid, adsorb onto the poly(*N*-isopropyl acrylamide) gel at a temperature above its LCST and desorb at a temperature lower than the LCST (Figure 3c). The use of a metal-ion complex for interaction with a thermoresponsive hydrogel is the key idea in this adsorption process. The selective recovery of metal ions is possible through the selection of chelating agents. This process enables the separation and recovery of metal ions without a large environmental impact through hydrophobic interactions in the water phase, without the use of organic solvents. Tokuyama et al. demonstrated similar solid-phase extraction processes for indium-phosphoric type surfactant complexes [91] and heavy-metal ion–humic substance complexes [92].

### 4.4. Contaminant Uptake with Self-Assembling and Gelation

**Figure 4:**
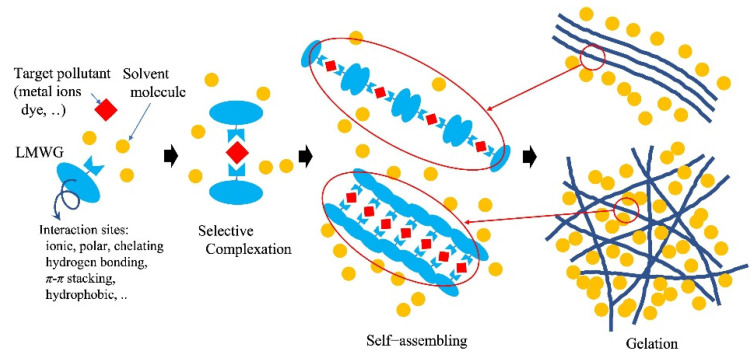
Example of metal ion removal by low-molecular-weight gelator (molecular gels).

Pollutants in an aqueous system can be removed by self-assembly of gelator molecules and gelation triggered by target substances using modern gelator molecules. This is similar to the adsorption and concentration of contaminants in the coagulation process using coagulants. In the case of Al-based coagulants, as many of the organic pollutants dissolved in water are negatively charged, the coagulants electrostatically couple with the pollutants, followed by coagulation to form gel-like flocks through hydrogen bonding among the aluminum hydroxides produced from the aluminum-based coagulants. The adsorption and removal of heavy-metal ions by polymers with gelation are similar to the method by which heavy-metal ions directly chelate with polymers to precipitate or aggregate. Chelating polymer coagulants adsorb metal ions via chelate bonding between the metal ions and ligands in the polymers. The suitable ligands for target metal ions depend on the type of metal ion (d10 type or transition metal type). The stability of the chelate complex depends on the balance between the covalency and ionic nature in the coordination bonding of the electron-donor groups in the ligands. High-performance coagulation occurs in the best metal ion–ligand combinations that neutralize the charge of the metal ion. Polymer coagulants efficiently form flocks and precipitate at the critical value of the zeta potential of the complex because of surface charge neutralization in the aqueous systems, achieving a high removal rate of metal ions. Therefore, it is important to control the solution conditions (pH, ionic strength, and solvent composition) to achieve the best adsorption performance. A similar mechanism is considered in the gelation system. Wang et al. (2016) reported the removal of Pb^2+^, Cu^2+^, and Cd^2+^ by the gelation of alginate through the direct binding of metal cations [42]. Small-molecule hydrogelators that selectively adsorb metal ions, anions, and dye molecules have been the focus of research in recent years. Small molecular gelators, so-called molecular gels and low-molecular-weight gelators (LMWG), produce self-assembled supramolecular polymer gels reversibly with non-covalent interactions, such as van der Waals forces, π-π stacking or hydrophobic effects, dipole–dipole, charge-transfer or coordination interactions, and hydrogen bonding (Figure 4) [106]. The gelators are highly solvated, responsive to external stimuli, show easy regeneration, offer an economic recovery, and exhibit rapid uptake of target adsorbates that are almost free from gel phase intra-diffusion. Lim et al. (2019) reviewed the recent development of molecular gels for remediation and wastewater treatment based on the type of gel and the mechanism of pollutant removal [65]. Sureshan et al. (2016, 2017) developed phase-selective organogelators by modification of 4,5-O-benzylidene-glycopyranosides to obtain hydrophobic supergelators of crude oil in water with small critical gel concentrations [87,107]. King and McNeil (2010) reported the highly selective in situ gelation of mercury using a low-molecular-weight gelator (Hg–quinoxalinone) [108]. Mercury is highly incorporated into the gel by in situ gelation with mercury acetate as the trigger for gelation, whereas the addition of chloride ions can easily desorb Hg. Das et al. (2021) introduced advances in the design, synthesis, and application of supramolecular gels made of phenylalanine and its derivatives [109]. They elucidated the self-assembly mechanisms, hierarchical structures, and gelation abilities of the phenylalanine derivatives. Patwa et al. (2015) demonstrated the removal of metal nanoparticles using a glycosylated-nucleoside fluorinated amphiphile in situ to trap <50 nm nanoparticles from contaminated water [110]. Hayes et al. (2012) developed a dye-gelling agent based on a urea core flanked by an aromatic nitro group and an aromatic diacid group [111], which uptakes methylene blue from several hundred to a 1000 mg/g. Molecular gels for the adsorption of inorganic anions are still few. This method requires precise molecular design for the gelling agent. Although its development is difficult, it is possible to develop a target-selective gelling agent for inorganic ions too.

### 4.5. Molecular and Ion Imprint Networks

**Figure 5:**
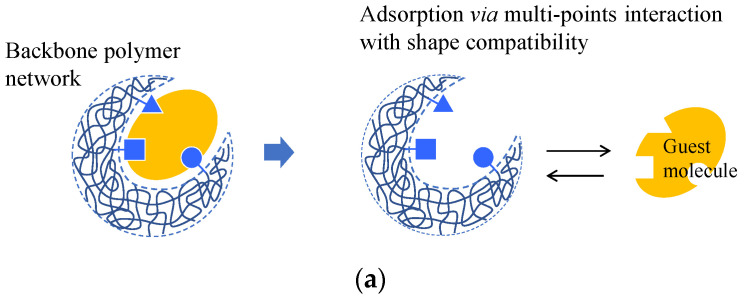

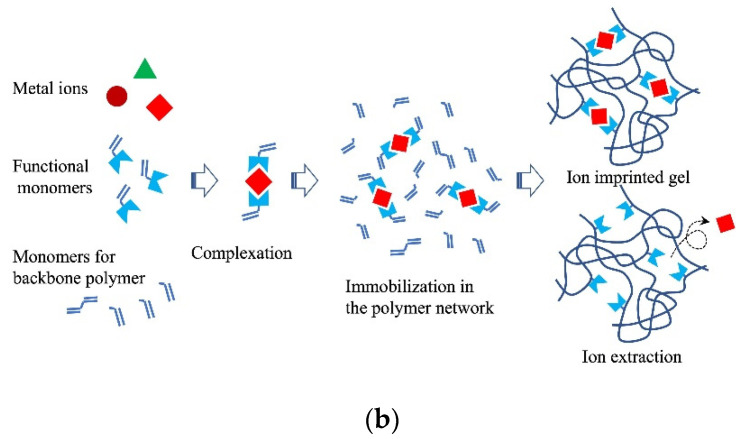
Schematic diagram of (**a**) molecular imprint and (**b**) ion imprint gels.

A complex between a molecule that uses a template and functional monomers is first formed during molecular imprinting. The monomers are then copolymerized with a crosslinking agent. The template molecule is then removed to obtain a molecular imprint polymer (MIP) with a binding space complementary to the template molecule (Figure 5a) [112,113]. Various methods have been proposed to improve the selective adsorption performance of imprinted gels. The fragment imprint method attains selective molecular recognition ability in the gel by using a part of the structure of the target molecule or a substance that is similar to the structure of a pseudo-template molecule (Figure 5a) [114,115]. The internal immobilization method uses a template molecule with an ionic functional group close to the target molecule. First, the complex between the template molecule and an ionic monomer that forms a complex with the template molecule is prepared. Then, a gel is synthesized in an organic solvent to dissolve the complex, second monomer, and crosslinking agent. After removing the template molecule, an imprinted gel with adsorption sites close to the target molecule is then obtained [116]. This technique enables the synthesis of imprint gels with target molecules that are hardly dissolved in any pre-gel solution.

Ion imprinting differs from molecular imprinting. Metal ions can be selectively separated using an ion-imprinted polymer gel. The metal salt of the target metal ion is first conjugated to the monomers of the gel. The conjugate is then polymerized with a crosslinking agent to obtain an ion-imprinted polymer gel with high selectivity (Figure 5b). Highly selective monitoring of metals (Hg(II), Pb(II), Cd(II), and As(V)) using ion-imprinted polymers was demonstrated by Pankaj et al. (2015) [66].

For molecular and ion-imprinted polymer gel adsorbents, it is essential to create pathways with easy access to the adsorption sites in the adsorbents. Li et al. (2014) reported a porous organic polymer (POP) with a high surface area and size-adjustable open pores as adsorbents for heavy metals [117]. This adsorbent, named nano-trap, exhibited high selectivity for Hg in its adsorption capacity (1 g-Hg of 1 g-Hg/1 g POP). Imprint gels using temperature-responsive polymer networks have also been developed to improve adsorption and desorption rates by controlling the swelling volume of the gel. Selectivity and adsorption capacity are trade-off factors. The adsorption capacity of imprint gels is small and its increase without decreasing the selectivity is an essential subject for imprint gels. In the case of macromolecules, mass transfer (diffusion) in the imprint gels is slow. Thus, improvement of the gel structure with access pathways to the imprinted sites is also crucial for rapid adsorption.

### 4.6. Reaction Field 

This type of gel utilizes chemical reactions in the gel to adsorb target substances effectively or effectively degrade the targets accumulated in the gel by adsorption. The gel serves as a field for the adsorption, accumulation, and reaction of the target substances. Over the past few decades, such gel applications have been developed as immobilization carriers for microorganisms and enzymes. In recent years, many studies have reported novel hydrogels with chemically reactive polymer networks or reaction assisting polymer networks, controllable inner environments, and highly dispersed catalytic materials.

**Figure 6:**
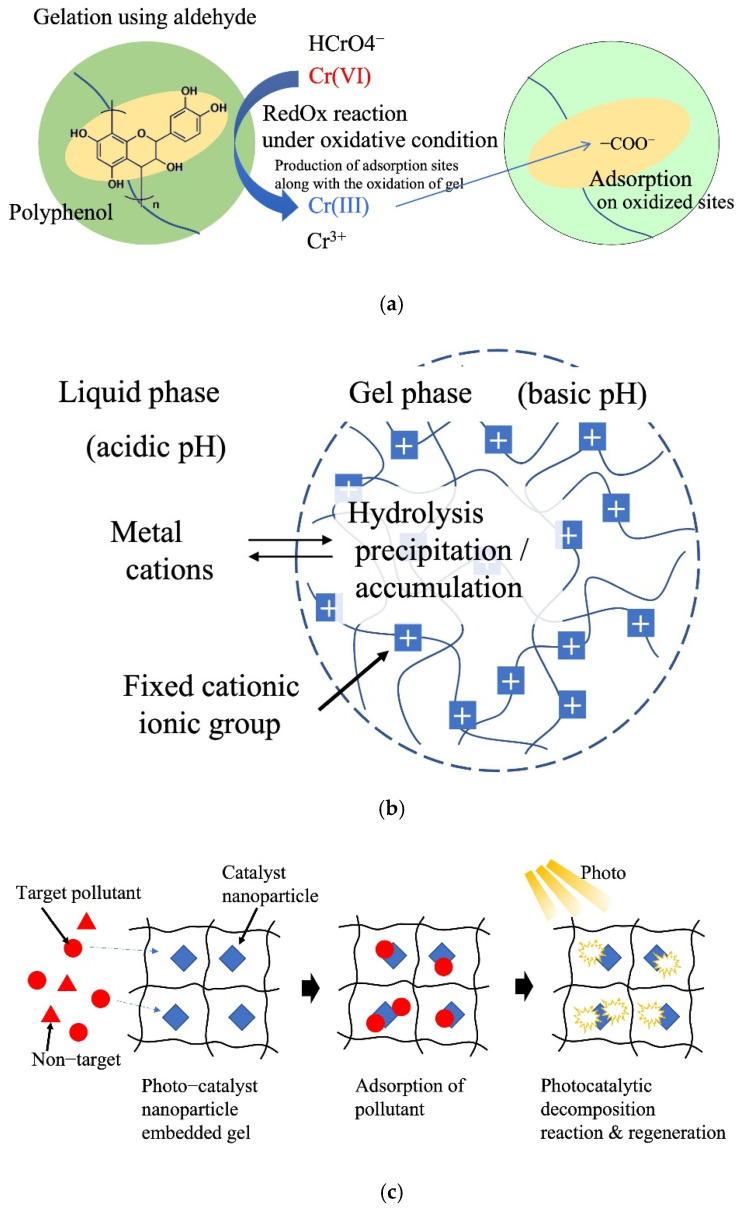
Reactive adsorption system. (**a**) Tannin gel redox adsorption system. (**b**) Hydrolysis of metal cations. (**c**) Adsorption and catalysis reaction.

Nakano et al. (2001) reported the adsorption and separation of hexavalent chromium using crosslinked condensed tannin gel [51]. Cr oxidizes tannin via a redox reaction to form a carboxyl group on the tannin gel. The Cr was reduced from Cr(VI) to Cr(III) by the tannin and adsorbed on the carboxyl and hydroxyl groups of the tannin gel via the ion-exchange method (Figure 6a). This reaction can be increased by adjusting the pH of the aqueous phase, enabling a large-scale removal of Cr (287 mg/g). The idea of incorporating a redox reaction mechanism into a gel to convert difficult-to-separate metal ions into easily adsorbable metal ions paves the way for developing a simple separation process that inculcates multiple separation processes within the gel particles. The redox potential of a natural polymer determines the availability of this reaction–adsorption system. Lü et al. (2021) reported a gallic acid-functionalized magnetic poly(ethyleneimine)/chitosan (PEI/CS) hydrogel for the enhanced synergistic reduction and adsorption of Cr(aq.) [118]. In this system, Cr(VI) is adsorbed via electrostatic interactions with positively charged amine groups of the gel. Gallic acid facilitates the reduction of Cr(VI) to less toxic Cr(III). Along with the reduction of Cr(VI), oxidation of phenolic hydroxy groups in the gel to carbonyl groups and low pH conditions due to gallic acid promote the adsorption of Cr(III) by chelating mechanism, resulting in a large adsorption capacity of 476.2 mg/g.

In the ionic gels of aqueous systems, the pH inside the gel becomes basic or acidic because of the Donnan equilibrium between the gel and outer liquid phase [119]. Utilizing this property of ionic gels, the precipitation of heavy-metal ions as hydroxides in the gel is possible. Gotoh et al. (2017) reported the recovery of metal ions through hydrolysis of metal ions in a cationic hydrogel using poly(N-[3-dimethylaminopropyl]acrylamide) gel (Figure 6b) [120]. The pH of the gel is controlled by varying the type and concentration of ionic groups. This system enables the immobilization of a large number of metal ions in gels in the form of poorly soluble hydroxides. Application for the remediation of soils contaminated with heavy-metal ions using this in situ immobilization has been proposed.

In recent years, many hydrogel adsorbents with highly dispersed and fixed photocatalytic nanoparticles have been reported. The adsorption and photocatalytic functions of the nanoparticles were applied to water and wastewater treatment. Yang et al. (2017) developed a composite hydrogel catalyst composed of poly(hydroxyethyl acrylate/N-methyl maleic acid) and CuS nanoparticles [121]. The gel adsorbed and concentrated sulfamethoxazole (SMX) antibiotics in the composite hydrogel. Subsequently, 90% of SMX was degraded upon visible-light irradiation by the photocatalytic reaction with embedded CuS nanoparticles (Figure 6c). Zhang et al. (2018) reported Fe_3_O_4_ nanoparticle-embedded porous hydrogel beads as catalysts for the Fenton reaction of methyl orange [31]. Zhu et al. (2021) reported poly(triethylene glycol methyl ether methacrylate) hydrogels as carriers of phosphotungstic acid for acid catalytic reactions in water [122]. Tokuyama et al. (2022) developed TiO_2_ nanoparticle-loaded poly(NIPA) fibers for the adsorption and photocatalytic degradation of organic pollutants in aquatic media [123]. 

### 4.7. Dissociation Control of Functional Groups in the Polymer Network

**Figure 7:**
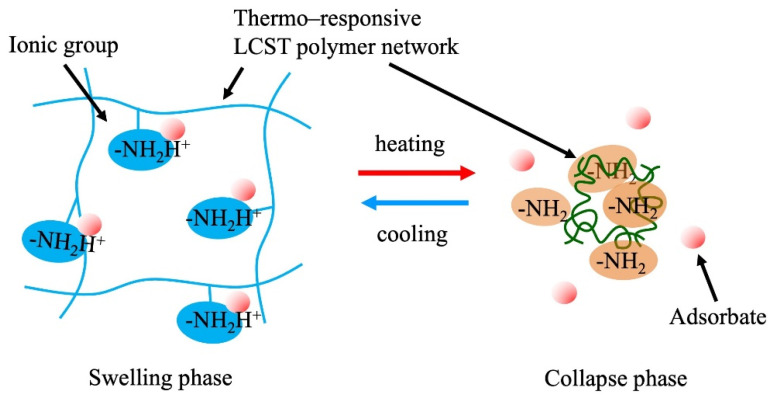
Adsorption control via p*K*a shift in the stimuli-responsive ionic hydrogels.

Changes in the dissociation of ionic groups on the polymer networks occur depending on the volumetric change in the crosslinked polymer hydrogels. Weak acid-type or weak base-type ionic groups on the polymer chains undergo a shift in their dissociation equilibrium p*K*a, with changes in pH inside the gel when the density of the ionic groups in the gels changes with the swelling or collapse of the gel (Figure 7). This phenomenon is considered to occur because of various phenomena, such as a change in the dielectric constant, counter ion condensation, and dehydration of the polymer. By utilizing this property, the adsorption control of target ions (the counter ions of the ionic groups in the gels) is achieved through volumetric swelling control of the gels. Thermoresponsive polymer gels are one of the candidates for this application because of their simple and easy volume control. Gotoh et al. (2010) reported the temperature-swing adsorption of phosphoric acid by poly(*N*-isopropyl acrylamide-*co*-*N*-[3-dimethylaminopropyl] acrylamide) copolymer hydrogels [124]. Honda et al. (2021) synthesized *N*-[3-(dimethylamino)propyl]methacrylamide (DMAPM)-*co*-*N*-*tert*-butyl acrylamide (TBAM) nanogels and applied them for the separation of carbon dioxide [125]. Nagasawa et al. (2019) reported the influence of the hydrophobicity of the backbone polymer on the thermoresponsive adsorption and recovery of carbon dioxide in a poly(*N*-isopropylacrylamide-*N*-*tert*-buthyacrylamide) copolymer gel slurry system [126]. A molecular design that produces a larger p*K*a shift is expected to yield a high-performance gel. To use this property, the mechanical strength of the gels to withstand repeated swelling operations is required. In addition, the gels must be micronized for a rapid response and adsorption. This process is energy-consuming when thermoresponsive gels are used because of the temperature-swing operation of the entire system. Therefore, it is crucial to devise methods to reduce energy consumption in practical applications. Effective utilization of the heat generated in chemical factories, steel mills, incinerators, etc. would increase the feasibility of these processes. Thin membranes fabricated on porous supports with nanosized particles of TBAM gels successfully achieved the energy-saving separation of carbon dioxide [125].

### 4.8. Diversification of the Application

Composite or conjugated forms of hydrogel adsorbents can increase the performance of the gel and the availability of the gels as adsorbents, considering the advantages of their use, cost, and material sharing from an ecological point of view. Adsorbents that exhibit advanced functions have been developed based on composite design. An example is the hydrogel embedding of magnetic nanoparticles [71]. Solid–liquid separation of fine adsorbent particles is easily performed using magnetic force in a liquid-phase separation system. Rehman et al. (2016) reported Fe_3_O_4_ nanoparticle-immobilized poly(3-acrylamidopropyl trimethylammonium chloride) microgels for the removal of AsO_4_^3−^ [127]. The magnetic field-responsive microgels were easily recovered from aqueous media for reuse. Because the adsorbents are lost over repeated use, the design of gels with a highly stable magnetic recovery rate is the subject of this study. In the application of spilled oil recovery, Xu et al. (2018) reported that poly(vinyl alcohol)/cellulose nanofiber (PVA/CNF) oil adsorbent gels with Fe_3_O_4_ nanoparticles effectively absorb oil spreading on the water surface, and the gels can be easily collected from the surface by the application of a magnetic field [128]. 

**Figure 8:**
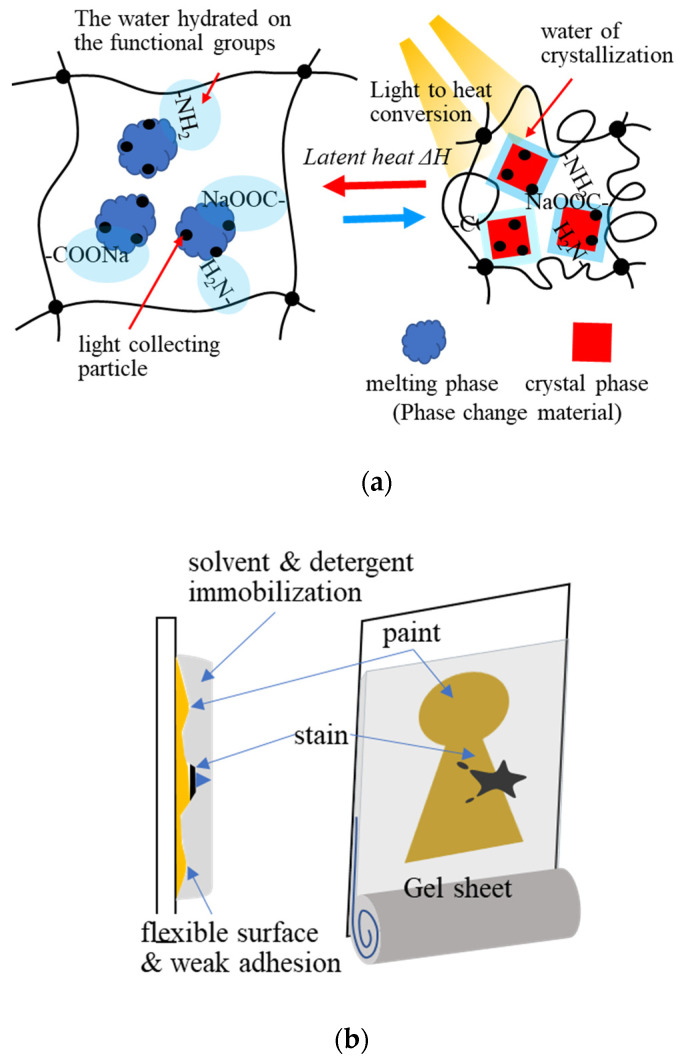
Application of the hydrogel adsorbents: (**a**) heat accumulation with light-to-thermal conversion, (**b**) conservation science: schematic image of the gel cleaning for painting art restoration.

Xiao et al. (2018) developed light-to-thermal conversion phase-change composite hydrogels consisting of sodium acetate trihydrate (NaAc3H_2_O), acrylamide–acrylic acid sodium copolymer, and CuS for the storage of solar heat energy by light-to-thermal conversion (Figure 8a). The composite hydrogel exhibited good energy conversion in a stable form during the phase change of NaAc3H_2_O. It restricted molecular water movement via hydrogen bonding with the functional groups on the polymer network of the gel, when NaAc3H_2_O was melting, without water leakage [129].

Applications of gel adsorbents have diversified in recent years. The adsorption and absorption properties of hydrogels have been applied in various fields. One of the applications of hydrogels is dressing materials [130]. Hydrogels can maintain a humid environment, absorb exuded tissue fluid, and exchange gas; making them a potential wound dressing material. Clean moist healing that adheres to the skin, absorbs exudate, disinfects the site, releases drugs through wound dressing, through fine particles of hydrogels are the scope of this application. In conservation science, in the 1980s, Richard Wolbers developed a “gel cleaning method” using gels such as gellan gum, agarose, and Pemulen TR-2 to restore and clean cultural properties [131,132,133,134]. In the gel cleaning method, stains on cultural properties are transferred to the gel. It is remarkably effective for cleaning the vertical surfaces of artworks such as paintings (Figure 8b). Gel cleaning with less volatilization of organic solvents (acetone, hexane, etc.) is also helpful for preserving the working environment. Hydrogel functions, such as solvent immobilization, surface flexibility, adhesion, adsorption, absorption, and extraction, are used in this technique. The multifunctional properties of hydrogel adsorbents can be further applied in different fields.

## 5. Perspective

The stimuli-responsive functions of gels during adsorption are of academic interest. One of the advantages of stimuli-responsive hydrogels is their tunable adsorption capacity by varying the stimuli of the gel. The highest adsorption capacity with optimum interaction distance between the target and functional groups of the gel was achieved by adjusting the swelling volume of the gel. Applications of stimuli-responsiveness in adsorption require proper control of the stimuli-responsive functions when considering industrial-scale applications. In fact, a lot of hydrogel adsorbent processes with stimulus-response functions have been proposed so far, but few of them have been used at the industrial level. To utilize the above-mentioned functions of the gel for easy and effective adsorption separation at the industrial level with lower costs, it is necessary to propose effective working methods for the adsorbent, such as the method of application, reactor type, and operational procedure of the adsorption separation process.

LMWG are very attractive and are expected to be more difficult to design than polymer hydrogels. Efficient gelation and recovery of pollutants using gelators are also important issues that need to be examined extensively. Because pollutants are taken in and gelled by forming molecular aggregates, the mechanical strength of the resultant aggregates is weak in principle, and further studies are required to reinforce their handling, through simple but complete recovery methods. In recent years, an increasing number of studies have proved this principle, but owing to the cost of its application, all parameters of this technique should be carefully studied before use. It is also advised to consider a combined usage of techniques, to make the best use of their specific characteristics.

The micronization of gel adsorbents is effective for quick adsorption and easy recovery. The adsorption sites in the gel could be effectively used owing to their large surface area with little diffusion inside the gels. Alternatively, it is effective to magnetize the gel to facilitate this or to impart a stimulus-response function that brings about a hydrophobic change in the gel for easy and simple recovery of the gel as a form of solid separation.

Because a large amount of adsorbent is used when adopting the adsorption method for environmental remediation such as water bodies polluted by oil spills and toxic chemicals, not only the applicability of the adsorbent but also the cost, simplicity of the recovery process, and design of a reusable adsorbent are critical issues for the consideration of adsorbent. The challenge for remediation is the design of recyclable and cost-effective adsorbents. 

Renewable natural polymers offer the advantages of low cost, abundance, and biodegradability for adsorbent applications. The use of natural polymers would be developed further in various fields, as is represented by the diversified applications of cellulose nanofibers in composite gels, and the adsorbent made from natural polymers is expected to be easier to use with the improvement of their performance. Considering the materials that meet these requirements at the industrial level, cellulose is one of the promising abundant, ecological, and raw materials for the adsorbent, and expanding the use of cellulose seems to be advantageous and competitive. Different from the conventional immobilized catalysts with porous support materials, the gel network has characteristic functions such as immobilizing a large number of nanoparticles with high dispersion, small mass transfer resistance, and controllable specific adsorption as introduced in this review. This diversity of adsorbent design is highly valuable and intensive developments are expected.

## 6. Summary

This paper reviews polymer hydrogels as adsorbents for removing pollutants in aqueous systems based on research papers published in the last decade. The characteristics and drawbacks of hydrogels were clarified from the perspective of adsorption separation technology. The relationship between the structures and functions of polymer networks was summarized, and the available functions of hydrogels for adsorbents and their designs were overviewed. The functions were classified in terms of the role and functions of the polymer network in adsorbent applications: (1) matrix for immobilization of nano- and microsubstances, (2) scaffold for the strategic immobilization of functional groups, (3) stimuli-responsiveness of the polymer network, (4) contaminant uptake by self-assembly and gelation processes, (5) ionic and molecular imprinting, (6) integrated reaction field, and (7) dissociation control of ionic groups via volumetric control of the gel. Hydrogels have the diversity of creating various adsorbents suitable for capturing potential pollutants using the available functions as indicated in this review. Hydrogel adsorbents have the potential to create a functional adsorption process with a small environmental impact by integrating multiple functions into the gels. The variety of structural designs and functions of gel adsorbents would continue to expand their application. This is an era of comprehensive design of adsorbents and adsorption processes. In the life cycle of an adsorbent, green technology based on both adsorbent design and the optimum adsorption process is required. However, this effort is still in its infancy.

## Figures and Tables

**Table 1:** Representative gel materials and involved functional groups.

Synthetic Monomer/Polymer [84,85]	Natural Polymer [48,53,54,64]
vinyl derivatives:	carrageenan (−OSO_3_^−^, −OH)
acrylamide (−NH_2_, >C=O)	alginic acid (−COOH, −OH)
derivatives:	pectin (−COOH, −OH)
methylpropane sulfonic acid (−SO_3_^−^)	gellan gum (−COO^−,^ −OH)
dimethyl aminopropyl (−N(CH_3_)_2_	
trimethyl ammonium (−N(CH_3_)_3_^+^ )	cellulose (−OH)
vinyl alcohol (−OH)	cyclodextrin (−OH)
acrylic acid (−COOH)	dextran (−OH)
styrene derivatives: −(C_6_H_6_)	
sulfonic acid (−SO_3_^−^)	chitin (>C=O, −NH, −OH)
poly(ethylene imine) (−N(CH_3_)_3_^+^)	chitosan (−NH_2_, −OH)
poly(ethylene glycol) (−O−)	
poly(aniline) (−N=, −(NH)−, −(C_6_H_6_))	biomass (polyphenol, −OH)

## Data Availability

Not applicable.
